# Type 2 diabetes in South Asians compared to Europeans: Higher risk and earlier development of major cardiovascular events irrespective of the presence and degree of retinopathy. Results from The HinDu The Hague Diabetes Study

**DOI:** 10.1002/edm2.242

**Published:** 2021-03-18

**Authors:** Judith van Niel, Petronella H.L.M. Geelhoed‐Duijvestijn, Mattijs E. Numans, Aan V. Kharagjitsing, Rimke C. Vos

**Affiliations:** ^1^ Department of Internal Medicine Haaglanden Medical Center The Hague The Netherlands; ^2^ Department of Public Health and Primary Care Leiden University Medical Center (LUMC Leiden The Netherlands; ^3^ Department Diabetology and Endocrinology University Hospital Brussels & Diabetes Research Centre Vrije Universiteit Brussel (VUB Brussel Belgium

**Keywords:** (time until) non‐fatal MACE, Retinopathy, South Asians, The HinDu The Hague Diabetes Study, type 2 diabetes

## Abstract

**Introduction:**

South Asians with diabetes have more severe diabetic retinopathy (DR) and cardiovascular complications than white Caucasians. However, how big this gap is and the relation with the severity of DR has not been studied. Here, we quantify the difference in time from diabetes diagnosis until a first non‐fatal Major Adverse Cardiovascular Event (TUF MACE) in different DR groups in South Asians and Europeans.

**Methods:**

3831 adults with type 2 diabetes, 1358 South Asians and 2473 Europeans, treated in our diabetes clinic between 2006 and 2017 were included. Data on risk factors, diabetes duration, age of diagnosis and diabetes complications were collected from the diabetes‐specific database and analysed using descriptive statistics and Cox regression. DR was graded in 3 categories, and non‐fatal MACE was pre‐specified.

**Results:**

Prevalence of non‐fatal MACE was the same when DR was absent, increased with increasing severity of DR in both ethnic groups, but was more frequent in South Asians with DR (mild: 50 vs. 42% and severe 62 vs. 46%. Classic risk factors only differed in relation to smoking habits, which were significantly lower in South Asians.

After correction for classic risk factors and age at diabetes diagnosis TUF MACE was significantly shorter in South Asians, an effect also seen in the no‐DR group (4.1 yrs. HR 1.5, 95% CI 1.3–1.8 and 7.4 yrs. earlier, HR 2.0, 95% CI 1.6–2.6 for no‐DR and severe DR, respectively).

**Conclusions:**

When adjusted for age at diabetes diagnosis, we show that time until first non‐fatal MACE in South Asians is significantly shorter compared to Europeans and increases from no‐ to severe DR.

## Graphical abstract

Time until first non‐fatal MACE in South Asians is 4 to7 years shorter compared to Europeans depending on the presence of retinopathy

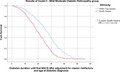

**What is already known**

1. South Asians have a higher prevalence of both diabetes and cardiovascular disease than white Caucasians at younger age
2. Diabetic retinopathy seems to be an independent risk factor for cardiovascular disease

**What this study has found**

Time from diabetes diagnosis until first MACE was found to be significantly shorter in South Asians compared to Europeans already in the no‐DR group and increases when diabetic retinopathy is present.

**Impact**

In South Asians, cardiovascular risk management demands an early aggressive approach of all cardiovascular risk factors to prevent MACE and narrow the gap in time until first MACE.

## 1 INTRODUCTION

The increased prevalence of both cardiovascular disease (CVD) and diabetic retinopathy (DR) in South Asians compared to white Caucasians has been reported in a number of studies. The Southall Diabetes Survey, an 11‐year follow‐up study, found that South Asians needed DR‐related laser therapy more often and earlier compared to Europeans (36% vs. 27%).^1^ South Asians were also found to have a three times higher risk for sight‐threatening retinopathy in one UK diabetes clinic, while they were on average 12.5 years younger in comparison with Europeans.^2^ Furthermore, in the Dutch AdRem study, the percentage of DR was 46% for South Asians versus 31% for Europeans.^3^ A similar difference, 45% versus 37%, was reported by the UK Asian DRP Study Group, accompanied by severe DR levels of 16 versus 12%.^4^ In cross‐sectional studies, diabetes duration, HbA1c, sex, the presence of macroalbuminuria, insulin therapy, systolic and diastolic blood pressure, cholesterol and younger age were all found to be independent risk factors for severe retinopathy.^2^, ^5^ Numerous studies have shown a relationship between DR and both CVD and cardiovascular mortality. Although DR is generally considered to be a microvascular complication, both the severity of retinopathy and its progression are known to be determinants of incident cardiovascular outcomes, underlining the importance of its presence.^6^, ^7^, ^8^, ^9^, ^10^ Furthermore, it has been suggested that changes in the retina reflect changes in other vascular beds, for example in the heart, kidney and brain, whilst people with the most rapid progression in retinal pathology may be the ones most likely to suffer incident CV outcomes. This hypothesis is further supported by data from Alonso et al. which showed that patients with type 2 diabetes and DR, who are free of clinical atherosclerotic disease, already have an increased number of atherosclerotic plaques in the carotid artery, a known risk factor for myocardial infarction and stroke.^11^ A recent cohort study from the UK found that developing type 2 diabetes at a younger age is associated with the occurrence of unfavourable classic risk factors. Counterintuitively, these unfavourable classic risk factors were twice as often present in whites compared to South Asians.^12^

Since South Asians generally develop diabetes and associated complications at a much younger age than white Caucasians, one could speculate that the higher prevalence of MACE found in South Asians is merely a result of the younger age of diabetes onset in combination with more severe retinopathy. Banerjee et al. reported remarkable data in this context, showing that middle‐aged South Asians with type 2 diabetes are at higher risk for biological ageing, a fact that may also predispose this group to various age‐related complications at a much earlier age.^13^ Together, these data suggest that excess cardiovascular risk in the South Asian population should also be sought beyond classic cardiovascular risk factors.

Given the above, we analysed time from diabetes diagnosis until a first nonfatal Major Adverse Cardiovascular Event (MACE), stratified for severity of DR, in a combined cohort of South Asians and Europeans, thereby including the effect of younger age of diabetes onset.

## 2 MATERIALS AND METHODS

### 2.1 Settings and population

The ancestors of the Surinamese Hindustani population in the Netherlands originally came from British India and were sent as contract workers to Suriname, a former Dutch colony. In medical literature, they are regarded as part of the South Asian population. With the independence of Suriname in 1975, many settled in the Netherlands, mainly in The Hague city area and its surroundings. For the purposes of this study, we only used data on participants with South Asian ancestors, that is migrants from Suriname, India, Pakistan, Bangladesh and Sri Lanka, and data from Europeans (of native Dutch origin and with both parents born in the Netherlands).

Participants ≥18 years treated for type 2 diabetes in the Hague Medical Centre diabetes clinic between 2006 and 2017 were selected from the diabetes‐specific electronic medical record (DiabetesNed) and categorized by country of origin. DiabetesNed was developed by AGG Solutions in the Netherlands using the DEMS format, with permission from the Mayo Clinic.^14^ Follow‐up time for each participant was calculated as time from diabetes diagnosis to date of event or date of censoring (death, referral to primary care, moving to another city/hospital).

Patients treated for other forms of diabetes, that is gestational diabetes, type 1 diabetes, steroid‐induced diabetes, and patients with active cancer were excluded.

### 2.2 Data collection and quality assessment

The year of diabetes diagnosis and year of diabetes complications per participant were extracted from the electronic medical record. A cross‐check was performed using the medical history in the hospital electronic medical record. Quality assessment of the cohort was performed following the STROBE/RECORD checklist for non‐randomized case‐control cohort studies ^15^

### 2.3 Outcome

Primary outcome was time (from diabetes diagnosis) until first (TUF) non‐fatal MACE.

TUF MACE was defined as the time from diabetes diagnosis to the development of either non‐fatal myocardial infarction, coronary artery bypass, percutaneous coronary intervention, non‐fatal stroke, peripheral artery surgery or percutaneous transluminal angioplasty.

Secondary outcome was the relationship between DR and TUF‐MACE.

### 2.4 Retinopathy

Diabetic retinopathy was defined as either 1) no, 2) mild or moderate only (non‐proliferative retinopathy) or as 3) severe retinopathy (proliferative, laser coagulation treatment, macula oedema).

### 2.5 Cardiovascular risk factors

Hypertension was defined as persistent systolic blood pressure of 140 mm Hg or above and/or treatment with antihypertensive medication and/or registered as hypertension in the medical record. Dyslipidaemia was defined as an LDL cholesterol of ≥116 mg/dl or was extracted from the patient record when treated as primary prevention. Smoking was documented using three categorical variables: current, previous smoker or never smoked.

### 2.6 Statistical analysis

Descriptive statistics including counts, percentages, means and standard deviations were used to describe patient characteristics. The chi‐squared test (for categorical variables) and the independent samples t test (for continuous variables) were used to assess differences between groups and expressed as *p*‐values (*p* < .05, regarded significant). Cox Proportional Hazards models were used to examine the differences between South Asians and Europeans in terms of TUF MACE in the three retinopathy subgroups (no, mild/moderate only or severe). Patients with a MACE before diabetes diagnosis were excluded from the analysis.

Three models were built, consisting of a crude model that accounted only for ethnicity, a second model that additionally included classic CV risk factors (hypertension, dyslipidaemia, smoking and sex) and a third model which further added age at diabetes diagnosis. Results are presented as hazard ratios (HR) with 95% confidence intervals (CI). SPSS 26 was used for all data analyses.

## 3 RESULTS

Patient characteristics of the two groups, further divided by sex are shown in Table 1. In the South Asian group, 92,6% are Hindustani and born in or had both ancestors from Suriname. The mean follow‐up time was 17.1 ± 10.8 years for South Asians and 15.1 ± 9.7 years for Europeans. Regarding age at diabetes diagnosis, South Asians were 10 years younger than the European subjects (42 ± 11.5 years vs. 52.9 ± 12). Within all DR groups, diabetes duration was comparable between both ethnicities. The percentage of participants with severe DR was twice as high in South Asians (26.5% versus 12.3%) In terms of the classic CV risk factors, both ethnicities showed approximately equivalent rates of hypertension and dyslipidaemia only differed significantly between men and women in the South Asian group. The percentage of participants who had never smoked, however, was significantly higher in South Asians (73.7% versus 56.3%) and was higher in women compared to men in both ethnic groups. All components of MACE, with the exception of stroke and peripheral vascular interventions, were more prevalent in South Asians and in men compared to women in both ethnic groups.

**TABLE 1:** Patient characteristics and possible confounding risk factors by ethnicity (N = 3831)

Patient characteristics	Total population		Men		Women	
	Europeans	SA	Europeans	SA	Europeans	SA
Number (%)	2473	1358	1344 (54.3)	654 (48.2)	1129 (45.7)	704 (51.8)
Age Diagnosis DM(years/SD)	52.9 ± 12.1	42 ± 11.5	52.4 ± 11.6	42.8 ± 11	53.5 ± 12.6	41.6 ± 12
Severe diabetic retinopathy n(%)	303 (12.3)	360 (26.5)	149 (11.1)	168 (25.7)	154 (13.6)	192 (27.3)
Smoking status never smoked n(%)	1392 (56.3)	997 (73.4)	647 (48.1)	414 (63.9)	745 (66.0)	583 (82.8)
Hypertension n(%)	1507 (60.9)	784 (57.7)	820 (61)	374 (57.2)	687 (60.9)	410 (58.2)
Dyslipidaemia n(%)	1010 (40.8)	596 (43.9)	543 (40.4)	306 (46.8)	467 (41.4)	290 (41.2)
History of myocardial infarction n(%)	510 (20.6)	345 (25.4)	329 (24.5)	204 (31.2)	181 (16.0)	141 (20.0)
History of CABG n(%)	282 (11.4)	239 (17.6)	193 (14.3)	139 (21.3)	89 (7.9)	100 (14.2)
History of PCI n(%)	357 (14.4)	291 (21.4)	219 (16.3)	166 (25.4)	138 (12.2)	125 (17.8)
History of stroke n(%)	342 (13.8)	234 (17.2)	197 (14.7)	118 (18.0)	145 (12.8)	116 (16.5)
History of carotids endarterectomy n(%)	34 (1.4)	11 (0.8)	24 (1.8)	5 (0.8)	10 (0.9)	6 (0.9)
PTA/peripheral vascular surgery n(%)	199 (8.0)	56 (4.1)	125 (9.3)	32 (4.9)	74 (6.6)	24 (3.4)

Abbreviations: CABG, Coronary Artery Bypass Graft; DM, Diabetes Mellitus type 2; PCI, Percutaneous Coronary Intervention; SA, South Asians.

### 3.1 Retinopathy and MACE

**FIGURE 1:**
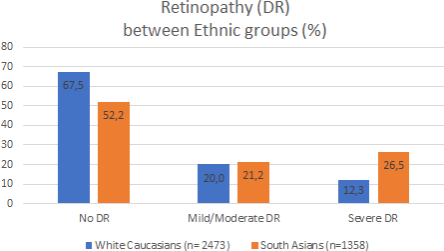
Prevalence of diabetic retinopathy in both ethnic groups

**FIGURE 2:**
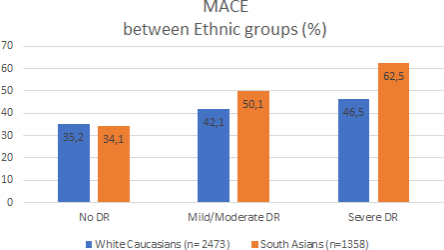
Percentage of MACE in different DR groups

Results of the percentage of participants with MACE and the classic risk factors per DR group are shown in Table 2. In both ethnic groups, DR was more prevalent as diabetes duration increased. Diabetes duration in the three DR groups did not differ between South Asians and Europeans. A higher percentage of European participants showed no DR (67.5 versus 52.2%, *p* < .01), whereas the percentage of severe DR was higher in South Asians (26.5 versus 12.3%, *p* < .01), see Figure 1. Of the classic CV risk factors, the percentage of participants who never smoked was higher in South Asians of all ages and DR groups. Hypertension was only significantly more prevalent in Europeans compared to South Asians in the group without retinopathy. Dyslipidaemia was significantly higher only in South Asians in the severe DR group. The percentage of cases developing MACE was higher in all participants with DR than without DR and significantly more prevalent in South Asians, see Figure 2.

**TABLE 2:** MACE and risk factors per ethnicity and DR group

Ethnic group	No retinopathy			Mild/Moderate retinopathy only			Severe retinopathy		
	Europeans	SA	*p*‐value	Europeans	SA	*p*‐value	Europeans	SA	*p*‐value
All Patients n(%)	1675 (67.5)	709 (52.2)	<.01	494 (20)	288 (21)	.63	303 (12)	359 (26)	<.01
Mean age at diabetes diagnosis/sd	53.9/12.1	42.4/11.5	<.01	51.4/11.5	43.9/11.2	<.01	49.8/11.9	39.7/11.5	<.01
Diabetes duration all patients yrs/m±sd	12.6 ± 8.9	13.3 ± 9.8	.54	15.6 ± 8.9	14.8 ± 8.5	.23	18.2 ± 9.9	18.7 ± 9.7	.88
MACE all patients n(%)	589 (35.2)	242 (34.1)	.76	208 (42)	145 (50)	<.01	139 (46)	224 (62)	<.01
Never Smoked all patients n(%)	926 (55.3)	515 (72.6)	<.01	293 (59.3)	215 (74.7)	<.01	173 (57.1)	266 (73.9)	<.01
Hypertension all patients n(%)	980 (58.8)	358 (50.5)	<.01	324 (65.6)	180 (62.5)	.34	202 (66.7)	245 (68.1)	.70
Dyslipidaemia total population n(%)	656 (39.2)	288 (40.6)	0.45	232 (47)	134 (46.5)	.50	122 (40.3)	174 (48.3)	.04

Abbreviation: SA, South Asians.

**FIGURE 3:**
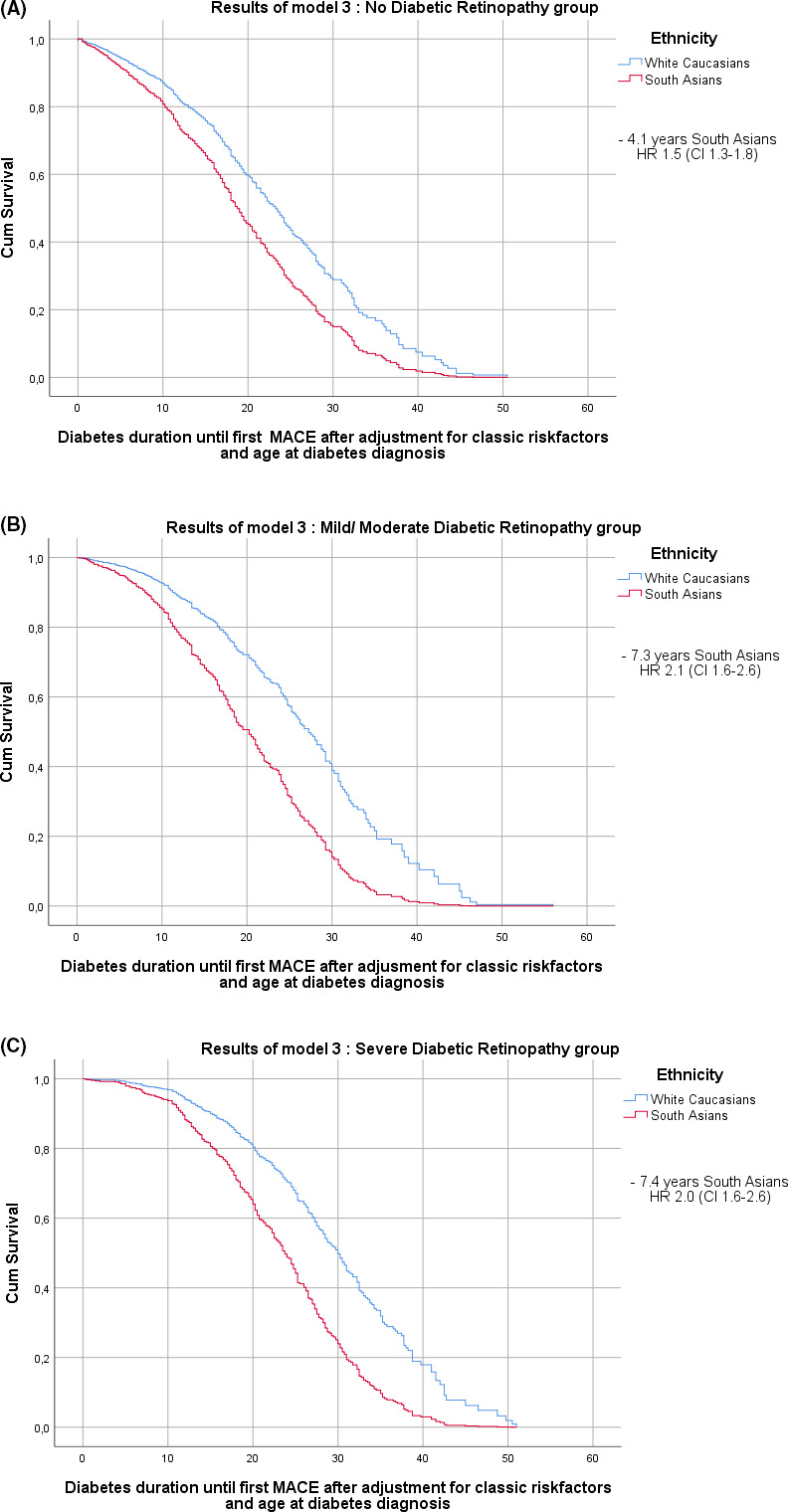
(A) Diabetes duration until first non‐fatal MACE in the no‐DR groups. (B) Diabetes duration until first non‐fatal MACE in the mild/moderate DR only groups

The results of time from diabetes diagnosis until first nonfatal MACE in the different DR groups are shown in Table 3 and Figure 3A‐C. In the no‐DR group, TUF MACE in Europeans was shorter than in South Asians (1.4 years HR 0.87 95%CI 0.8–1.0), but only in the crude Model. When corrected for classic risk factors and time at diabetes diagnosis (Model 3), South Asians developed their first MACE already 4.1 years earlier (HR 1.5, 95% CI 1.3–1.8) than Europeans.

**TABLE 3:** Diabetes duration until first MACE (TUF MACE)

Ethnic group	No retinopathy		Mild/Moderate retinopathy only		Severe retinopathy	
	Europeans	SA	Europeans	SA	Europeans	SA
Crude model^a^ TUF MACE (yrs) all patients	−1.4			−2.1		−2.1
HR TUF MACE all patients (95% CI)	0.87 (0.8–1.0)			1.2 (0.97–1.5)		1.2 (0.99–1.5)
Model 2^b^ TUF MACE (yrs) all patients	−0.9			−2.8		−1.9
HR TUF MACE all patients (95% CI)	0.9 (0.8–1.1)			1.3 (1.1–1.6)		1.2 (0.97–1.5)
Model 3^c^ TUF MACE in yrs all patients		−4.1		−7.3		−7.4
HR TUF MACE all patients (95% CI)		1.5 (1.3–1.8)		2.1 (1.6–2.6)		2.0 (1.6–2.6)

Abbreviations: SA, South Asians; TUF, Time until first MACE.

^a^ Crude model only ethnicity included.

^b^ Model 2 additionally including the classic risk factors (sex, hypertension, dyslipidaemia, smoking).

^c^ Model 3 = after adjustment for classic risk factors and age at diabetes diagnosis.

South Asians with mild/moderate retinopathy only developed their first MACE 7.3 (HR 2.1, CI 1.6–2.6) years earlier.

When severe retinopathy was present, South Asians developed their first MACE 7.4 years (HR 2.0 CI 1.6–2.6) earlier.

## 4 DISCUSSION

In our cohort of more than 1300 South Asians, time from diabetes diagnosis until first nonfatal MACE was significantly shorter compared to Europeans, already in the group without DR, although the overall percentage of participants with a MACE in the no‐DR group was comparable between the two ethnic groups. This finding is especially striking taking into account the much higher percentage of persons that never smoked in the South Asian group. The difference in TUF MACE between the two ethnic groups did not further increase with increasing DR severity. Differences in glucose regulation may explain the higher prevalence of severe DR in South Asians. Since diabetes duration is comparable in both ethnic groups, we do not think that glucose regulation explains the large difference in TUF MACE already seen in the no‐DR group. We also know from previous studies that the impact of glucose regulation on macrovascular complications is relatively small. For example, the INTERHEART study reported a population attributable risk for diabetes of 9.9%.^16^ We do find the same relationship between severity of DR and more MACE as has been demonstrated in many studies that also stressed its implications for care.^6^, ^7^, ^8^, ^9^, ^10^ In the ACCORD EYE study^6^ for example, a clear relationship was found between the progression of DR and the occurrence of incident CV events at 4‐year follow‐up, with a HR of 1.49 for mild and 2.35 for severe DR compared to no DR. In our study, the prevalence of MACE in European participants increased from 35.2% in the no‐DR group to 46% in the severe DR group (OR 1.3), whereas in South Asians it increased from 34.1 to 62% (OR 1.82) in the equivalent groups. Clearly, the development of microvascular disease, that is DR, further increases the cardiovascular risk profile, as was also shown in the UK‐DRIVE study,^17^ effectively compounding risk. The higher prevalence of MACE in South Asians is generally attributed to the younger age at diabetes diagnosis.^18^ However, we showed that, when corrected for age at diabetes diagnosis, South Asians still had a shorter TUF non‐fatal MACE than Europeans already in the no‐DR group.

Several valuable contributions have been made to the subject of the markedly unfavourable CV risk profile of South Asians, one of which is the INTERHEART study.^16^ Although smoking behaviour and elevated ApoB/ApoA1 ratio were determined to be the most important CV risk factors and together explained two thirds of total risk, South Asians were significantly less likely to be smokers compared to Europeans a finding replicated in our study. Moreover, several other studies showed excess risk factors for the development of CV complications in South Asians like high levels of Lp(a), low levels of protective adiponectin already observed in babies of 3 to 6 months old, suggesting that South Asians are probably exposed to atherogenic factors at very young age.^18^, ^19^, ^20^

A hopeful development is that the difference in CV morbidity and mortality between South Asians and Europeans declined after 2000, following the introduction of statins as a standard primary prevention therapy for patients with type 2 diabetes.^21^ Since statins are currently widely used but do not lower apoB, further options include even stricter LDL lowering or the use of the new PCSK9 inhibitors that specifically lower apoB. These new strategies may help in further narrowing the difference in TUF MACE between South Asians and Europeans. Promising data demonstrating the potential of this new class of lipid‐lowering drugs comes from the recently published FOURIER trial. In this trial, the investigators showed that the PCSK9 inhibitor evolocumab achieved an impressive additional reduction in cardiovascular risk in a high‐risk study population, even after only 2.2 years of follow‐up.^22^

When interpreting the results of the current (retrospective observational) study, several limitations should be considered. First, our findings are based on a single‐centre hospital‐based cohort study. Nevertheless, we believe that our results are representative, since most South Asian Hindustani in the Netherlands live in the area of the Hague and most of them with diabetes are treated in our clinic. All included participants were referred to our diabetes clinic due to insufficient glucose control and/or diabetic complications, came from the same region and had a mean follow‐up of 16 years since diabetes diagnosis. Although most studies on this subject are performed in first‐line settings (ie community or general practice‐based), most of our data were either in line or comparable with international data. In addition, our use of double‐staged data collection reduced the risk of missing data. Secondly, not all participants entered the study at the same time, but this was accounted for in the statistical analysis. Thirdly, we appreciate the heterogeneity within the South Asian population as pointed out by Bhopal et al.^23^ However, even though participants from several South Asian countries were included, 92.6% of our patients were South Asian Hindustani from Suriname, suggesting that our group had limited heterogeneity. Finally, given the nature of our data set, we did not analyse the temporal changes suggested by some, even though temporal shifts, that is a lower CV risk profile in the younger generation, may well exist.^21^

## 5 CONCLUSIONS

This is the first study to quantify the difference in TUF non‐fatal MACE between South Asians and Europeans after adjusting for classic CV risk factors and age at diabetes diagnosis, an effect that is already present in the absence of DR.

Our data support previous reports on the higher prevalence and severity of DR in the South Asian population and the relationship between DR severity and MACE. However, although the presence of DR is rightly seen as an important CV predictor, it is important to realize that its absence does not reduce the higher CV risk found especially in subgroups like South Asians. Therefore, in South Asians, CV risk management demands an early aggressive approach of all CV risk factors to narrow the gap between South Asians and Europeans in terms of MACE and TUF MACE.

## CONFLICT OF INTEREST

All authors declare no conflict of interest related to the manuscript.

## AUTHOR CONTRIBUTIONS

JvN gathered data, conducted statistical analyses, wrote the first draft of the manuscript and discussed, reviewed and edited the manuscript. PHG gathered data. PHG, MEN, AK and RCV discussed, reviewed and edited the manuscript. JvN is the guarantor of this work and, as such, had full access to all the data in the study and takes responsibility for the integrity of the data and the accuracy of the data analysis.

## Data Availability

The data that support the findings of this study are stored in Castor Data Management system and available on request. The data are not publicly available due to privacy or ethical restrictions.
